# Genomic Characterization of *Aureimonas altamirensis* C2P003—A Specific Member of the Microbiome of *Fraxinus excelsior* Trees Tolerant to Ash Dieback

**DOI:** 10.3390/plants11243487

**Published:** 2022-12-13

**Authors:** Regina Becker, Kristina Ulrich, Undine Behrendt, Volker Schneck, Andreas Ulrich

**Affiliations:** 1 Microbial Biogeochemistry, Research Area Landscape Functioning, Leibniz Centre for Agricultural Landscape Research (ZALF), 15374 Müncheberg, Germany; 2 Institute of Forest Genetics, Johann Heinrich von Thünen Institute, 15377 Waldsieversdorf, Germany

**Keywords:** plant-bacterium interaction, genome mining, ash dieback, *Hymenoscyphus fraxineus*, microbiome

## Abstract

Some European ash trees show tolerance towards dieback caused by the invasive pathogen *Hymenoscyphus fraxineus*. The microbiome of these trees harbours a range of specific bacterial groups. One of these groups belonging to the species *Aureimonas altamirensis* was studied in detail by genome analysis and a plant inoculation trial. The strain group was shown to be phylogenetically distinct from clinical isolates by 16S rRNA analysis and phylogenomics. Genome analysis of a representative strain C2P003 resulted in a large number of unique gene sequences in comparison to other well-studied strains of the species. A functional analysis of the genome revealed features associated with the synthesis of exopolysaccharides, protein secretion and biofilm production as well as genes for stress adaptation, suggesting the ability of C2P003 to effectively colonize ash leaves. The inoculation of ash seedlings with C2P003 showed a significant positive effect on the plant health of the seedlings that were exposed to *H. fraxineus* infection. This effect was maintained over a period of three years and was accompanied by a significant shift in the bacterial microbiome composition one year after inoculation. Overall, the results indicate that C2P003 may suppress *H. fraxineus* in or on ash leaves via colonization resistance or indirectly by affecting the microbiome.

## 1. Introduction

The plant microbiome is an integral part of its host and comprises a tremendous amount of beneficial, commensal and potentially pathogenic microorganisms. Both plant-microbe interactions and competition among microorganisms affect the plant performance [1,2]. Plants subjected to diverse biotic and abiotic stresses can induce changes in root and leaf exudates and subsequently alter the plant microbiome assembly [3]. A special role in the microbiome is played by plant beneficial bacteria that effectively colonize the plant and contribute to nutrient supply [4,5] and/or defence against pathogens by direct antagonism, competition or plant-supporting metabolic activities [6,7,8]. The abundance of these microorganisms in the microbiome has been linked to plant growth and health in several studies [9,10,11]. The spread of pathogens may have dramatic effects on ecosystem functioning, particularly in forests. This makes it all the more important to successfully control the pathogens of the trees through the application of biocontrol agents [12].

In the last few years, the spread of ash dieback has resulted in a substantial threat to *Fraxinus excelsior* stands in central and northern Europe [13,14]. Infection with the causal agent *Hymenoscyphus fraxineus* begins with the germination of wind-borne ascospores on the leaves, after which the fungus penetrates into the intercellular space and then into shoots and twigs, where it causes necrotic bark lesions and blockages of the xylem vessels [15]. The severity of infestations with *H. fraxineus* might be reduced by resident phyllosphere or endosphere colonizers, which are able to counteract the entry and penetration of the invader [16,17]. Comparative analyses of the bacterial leaf microbiome of *F. excelsior* in ash dieback-affected forests revealed a significantly increased abundance of individual bacterial groups in tolerant trees belonging to the genera *Aureimonas, Luteimonas*, *Pseudomonas*, *Bacillus* and *Paenibacillus* [18]. With the exception of *Aureimonas*, all these genera are known for strains with plant-beneficial traits. Thus, a health-stabilizing effect on ash seedlings was recently demonstrated for a *Luteimonas fraxinea* strain obtained from the leaves of healthy ashes [19]. Other members of *Luteimonas* also had stimulating effects based on disease suppression and nitrogen acquisition [20,21,22]. The genus *Pseudomonas* comprises many strains contributing to plant health by antibiosis, competition and induction of systemic resistance [23,24,25,26]. Finally, strains of the genera *Bacillus* and *Paenibacillus* are of exceptional importance as biocontrol agents, especially due to the synthesis of bioactive secondary metabolites [27,28,29].

Unlike these genera, members of *Aureimonas* have not yet been characterized as plant-beneficial bacteria. At present, the genus contains 18 species originally derived from a wide range of habitats. *Aureimonas altamirensis* was first isolated from the subterranean environment of the Altamira cave as part of a complex microbial community [30]. In the following years, several publications reported on the pathogenic potential of *A. altamirensis* strains based on evidence in human clinical samples [31,32,33,34]. However, the clinical relevance of the species remains unclear since infestations are discussed in relation to immune deficiencies and the simultaneous detection of other organisms [32,35,36]. *A. altamirensis* is therefore considered an opportunistic pathogen [36,37], and further studies are needed to elucidate its epidemiology, pathogenic mechanisms, and clinical significance [38]. Moreover, the species was also repeatedly detected in environmental habitats, e.g., as a cellulolytic strain obtained from vegetable biomass [39].

We discovered *A. altamirensis* strains as specific colonizers of leaves from dieback tolerant ash trees [18] and hypothesized that the species could play a role in plant resilience. Here we characterize this group of isolates in detail. The phylogenetic analysis of a representative isolate was performed to determine the position within the genus and the species. The genome was further investigated to reveal features that could be related to plant colonization and plant-protective properties. Finally, an inoculation trial on ash seedlings exposed to *H. fraxineus* was performed to clarify the effects of the studied isolate on plant health and the microbiome.

## 2. Results and Discussion

Analyses of the leaf microbiome of 80-years-old ash trees under *H. fraxineus* exposure revealed several bacterial groups with significantly higher abundance in tolerant trees [18]. One of these groups was assigned to *A. altamirensis,* which was found together with well-known plant colonizers, such as *Luteimonas* and *Pseudomonas*. The abundance of the *A. altamirensis* strain group was increased 34 and 49-fold in tolerant trees as analysed by 16S rRNA gene amplicon sequencing and culturing, respectively. A total of 62 isolates formed this single group based on MALDI-TOF MS (matrix-assisted laser desorption/ionization time-of-flight mass spectrometry), which were isolated almost exclusively from tolerant ash trees.

### 2.1. Phylogenetic Analysis

**Figure 1:**
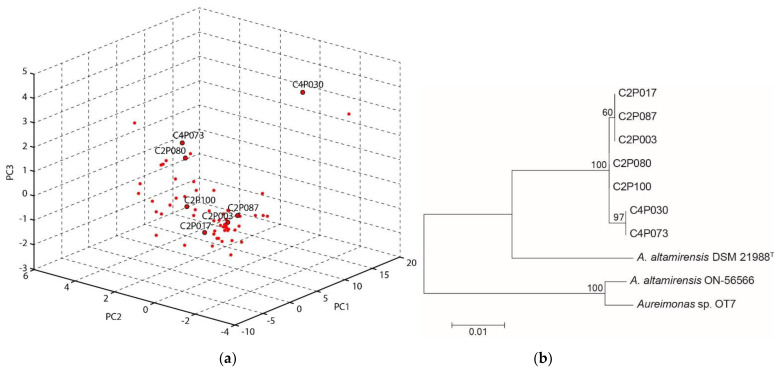
Variability of the *A. altamirensis* isolates obtained from ash trees: (**a**) Ordination plot of MALDI-TOF MS spectra, calculated by a principal component analysis. (**b**) Maximum-likelihood tree of the IGS gene sequences of selected ash-associated *A. altamirensis* isolates and two clinically derived strains (ON-56566, OT7) as well as the type strain DSM 21988. Numbers at branch nodes refer to bootstrap values >50%.

To gain insight into the variability of the ash-associated *A. altamirensis* group, peak profiles derived from MALDI-TOF MS spectra of the 62 isolates were analysed by principal component analysis. The ordination plot indicated clear differences between the isolates (Figure 1a). The isolate C4P030 showed a strong distance to nearly all other group members. In addition, several different clusters could be identified within the *A. altamirensis* strain group. Seven isolates with clear distances to each other were selected for sequencing of the ribosomal intergenic spacer (IGS) region. For phylogenetic analysis, the IGS sequences of the isolates were compared with those of the type strain DSM 21988 and the clinically derived strains ON-56566 and OT7. The tree shows a separate cluster of all the ash isolates. Within this branch, the isolates obtained from different trees (C2 and C4) could be distinguished. A strong differentiation was found to DSM 21988 and with even higher distances to the clinical strains ON-56566 and OT7 (Figure 1b). The sequencing of the 16S rRNA gene revealed identical sequences for all seven isolates. The isolate C2P003 was subsequently chosen as a representative isolate and subjected to a comprehensive analysis.

**Figure 2:**
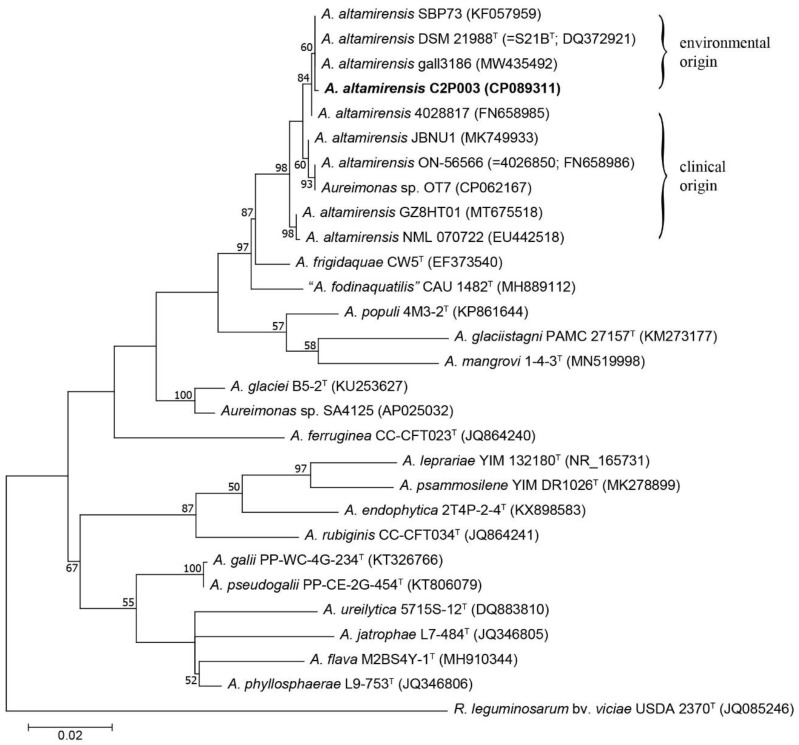
Maximum-likelihood tree of the 16S rRNA gene sequences showing the position of *A. altamirensis* C2P003 among other *A. altamirensis* strains and reference strains of related *Aureimonas* species. Phylogenetic analysis was performed using the HKY+G+I model. *Rhizobium leguminosarum* was used as the outgroup. Numbers at branch nodes refer to bootstrap values >50%. Bar: substitutions per nucleotide site. Accession numbers or locus tags (NCBI or IMG database) are indicated in brackets.

A phylogenetic analysis based on the 16S rRNA gene showed a close relationship of isolate C2P003 to the type strain DSM 21988 (isolated from the subterranean environment of a cave), the strain gall3186, which is a member of a gall wasp microflora [40], and SBP73, a cellulolytic strain obtained from vegetable biomass [39] (Figure 2).

The four strains of environmental origin formed a shared cluster that was distinct from the isolates detected in clinical samples [31,32,37,38,41]. Within the genus, *A. frigidaquae* and “*A. fodinaquatilis*” were found to be the closest related species. However, the phylogenetic positions of the *Aureimonas* species within the genus as suggested by the 16S rRNA gene analysis were only slightly supported by the bootstrap values. Consequently, the analysis was complemented by genome phylogeny using the genome sequences of the currently known *Aureimonas* species. The comparison between C2P003 and the type strain of *A. altamirensis* DSM 21988 resulted in an average nucleotide identity (ANI) of 96.4% and a digital DNA-DNA hybridization value (dDDH) of 70.0%. Both values were above the respective thresholds (≥95% and ≥70%) and confirmed the affiliation of C2P003 to the species *A. altamirensis*. Comparable to the 16S rRNA analysis, the phylogenomic tree showed the close relationship between C2P003 and *A. altamirensis* DSM 21988 with a clear separation to *A. altamirensis* ON-56566 and *Aureimonas* sp. OT7, supported by high bootstrap values (Figure S1). Strain OT7, isolated from human skin, and strain ON-56566, isolated from patient blood culture, were reported to belong to the same species clade that is separated from the *A. altamirensis* type strain [41]. Consistent with the 16S rRNA data, a phylogenomic analysis indicated that *A. frigidaquae* and “*A. fodinaquatilis*” were the species most closely related to *A. altamirensis*.

### 2.2. Genome Analysis

The sequencing and assembly of genomic DNA from the *A. altamirensis* strain C2P003 resulted in a complete circular genome. However, the annotation of the genome sequence revealed several clusters with plasmid genes (*trbIGFLJEDCB*, *repB*) (Table S1). It can therefore not be ruled out that in the current assembly, the chromosome was merged with one or several plasmids. Accordingly, the latest assembly (GCF_021228915.1) should be considered as a draft genome. The genome sequence comprised 4,592,981 bp with a total of 4289 predicted protein-coding genes (Table 1). The genome size, G+C content and the other annotation statistics are comparable to those of the *A. altamirensis* type strain DSM 21988 and the clinically derived strains ON-56566 and OT7. However, strain C2P003 differs from its closest relatives by a larger genome size and a higher number of protein coding sequences.

**Figure 3:**
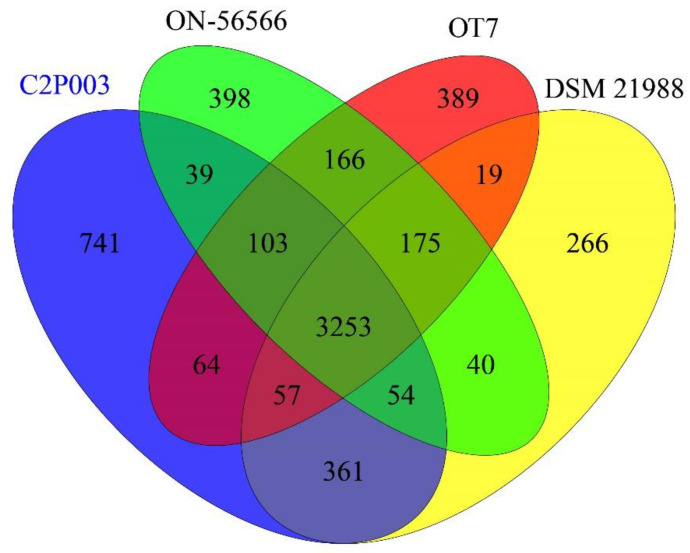
Venn diagram showing orthologous genes between the genomes of *A. altamirensis* C2P003, DSM 21988, ON-56566 and OT7.

The genomes of the four *A. altamirensis* strains share 3253 orthologous genes (Figure 3), which account for 69.6% of all predicted protein-coding sequences of strain C2P003. A total of 3725 genes of C2P003 (79.7%) had orthologues in *A. altamirensis* DSM 21988, indicating the close relationship between the two strains. By comparison, lower proportions of genes (73.28 and 74.4%) were shared with ON-56566 and OT7, respectively. In addition, the analysis of orthologous genes again revealed the specificity of C2P003 due to the highest quantity of unique gene sequences (741 vs. 266–398).

Genome annotation did not reveal any genes whose products are clearly associated with pathogenic functions. In addition, a comparison with pathogenic *Brucella* species that possess a set of virulence factors allowing bacteria to replicate in host cells and to induce persistent infections was performed. A key role in the virulence of *Brucella* is played by the T4 secretion system (T4SS), which is used to transfer toxic effector proteins into the host cell [42,43]. On the other hand, T4 secretion systems are equally common in nonhuman-pathogenic species, such as symbiotic rhizobia [44]. The genes for T4SS transfer proteins could also be detected in the genomes of the *Aureimonas* strains C2P003, DSM 21988 and ON-56566 (Table 2 and Table S1). Further virulence factors of *Brucella*, in particular genes involved in the biosynthesis of the lipopolysaccharide O-side chain (e.g., *wboA*, *wboB*, *wbkA*, *wbkE*, *gmd*) [45], could not be identified in the genome of any *Aureimonas* strains. The phenotypic tests with strain C2P003 did not indicate a pathogenic potential. The strain was not able to grow at human body temperature (37 °C), did not show extracellular DNAse activity and was haemolytic-negative. Overall, there were no indications for pathogenicity.

### 2.3. Genomic Features

To study the genetic bases for a plant-associated lifestyle and potential plant-enhancing properties of *A. altamirensis* C2P003, a functional analysis of the annotated genome was performed. The analysis, conducted in comparison to the type strain DSM 21988 and the clinical strain ON-56566, was focused on the detection of gene mediating functions in plant colonization, defence against competitive microorganisms and resistance to abiotic stress.

#### 2.3.1. Host Colonization

Bacteria gain access to plant tissues and cells by motility, chemotaxis, biofilms and the ability to degrade plant polymers [46,47,48]. Numerous genes conferring these traits have been identified in the genomes of plant-colonizing bacteria [49,50,51]. Cells of C2P003 as well as *A. altamirensis* DSM 21988 [30] were found to be nonmotile and genome analysis of the three *A. altamirensis* strains did not reveal genes conferring motility and chemotaxis. However, strains C2P003, DSM 21988 and ON-56566 possess several genes related to the synthesis of exopolysaccharides and protein secretion system types I, IV and VII (Table 2, Supplementary Table S1). The secretion of both polysaccharides and proteins promotes the attachment of bacteria to the host cells and thus the formation of biofilms [52,53]. Protein secretion systems, in particular, are involved in numerous biotic interactions, including mutualistic plant colonization but also the infection induction by pathogens [54,55]. In contrast to strains C2P003 and DSM 21988, the genome of ON-56566 lacks genes for secretion systems type I and VII.

Moreover, all analysed *A. altamirensis* genomes harbour genes coding for plant cell wall-degrading enzymes that are crucial for bacteria to be able to access and proliferate in the intercellular space [46,56]. The detected genes encode for several enzymes involved in the degradation of cellulose, hemicellulose and pectin (Table 3, Supplementary Table S2).

#### 2.3.2. Antimicrobial Substances

Some further genes in the genomes of C2P003, DSM 21988 and ON-56566 encode for enzymes required in the degradation of bacterial and fungal cell walls, such as β-*N*-acetylglucosaminidase, *N*-acetylmuramoyl-L-alanine amidase and phage lysozyme R (Table 3, Supplementary Table S2). β-*N*-acetylglucosaminidase plays an important role in the degradation of chitin, the bulk component of fungal cell walls [57], as well as in the degradation of peptidoglycan, the main component of bacterial cell walls [58,59]. *N*-acetylmuramoyl-L-alanine amidase is the second most important enzyme required in the lysis of peptidoglucan [58,60], whereas lysozyme contributes to the breakdown of peptidoglucan and partly also of chitin [61,62]. In addition, the three *A. altamirensis* strains are equipped with antibiotic resistance genes (Table 2). Both the ability to attack microbial competitors and the self-protection from invaders may support the establishment of C2P003 in the host.

#### 2.3.3. Stress Adaptation

Colonizers of the phyllosphere, the interface between the atmosphere and plants, are exposed to strong and unpredictable fluctuations in temperature, moisture and UV levels. Adaptations to osmotic and oxidative stress are therefore basic abilities for survival in this habitat [63].

The most common response to osmotic stress is the accumulation of potassium or osmolytes, such as amino acids, betaines, polyols or sugars [64]. The genome of C2P003 contains genes coding for betaine aldehyde dehydrogenase and choline dehydrogenase, which are involved in the biosynthesis of the osmoprotectant glycine betaine from choline [65,66] and the production of glycine betaine [67], respectively. Furthermore, the presence of the conserved *yehZYXW* operon encoding the stress-regulated ABC membrane transport system [68] might contribute to the resilience of C2P003 against osmotic stress. Genes related to the osmotic stress response were also found in the other *A. altamirensis* strains (Table 2, Supplementary Table S1).

To counteract oxidative stress and to restore a redox balance, aerobic organisms possess complex mechanisms regulating the activation or silencing of genes for defensive enzymes, transcription factors and structural proteins [69,70]. Genes coding for antioxidant enzymes, i.e., superoxide dismutase [71] and glutathione S-transferase [72,73], were found in the genomes of C2P003, DSM 21988 and ON-56566 in equal numbers (Table 2, Supplementary Table S1).

In addition to harmful atmospheric effects, heavy metals absorbed by the plants can also damage plant-associated bacteria. In response to this challenge, bacteria have evolved a range of mechanisms for heavy metal tolerance including transport, sequestration and reduction of metal ions [74,75,76]. This enables plant-associated metal-tolerant bacteria to reduce the metal contamination of plants and to enhance plant growth [77,78]. Genes contributing to copper homeostasis, cobalt-zinc-cadmium resistance and resistance to chromium compounds were detected in the genomes of C2P003, DSM 21988 and ON-56566 (Table 2; Supplementary Table S1).

In conclusion, the analysis of the genomic features of *A. altamirensis* C2P003 revealed numerous genes involved in metabolic functions for survival and competitiveness in the phyllosphere and the plant endosphere.

### 2.4. Inoculation of Ash Seedlings

The effect of *A. altamirensis* C2P003 on ash dieback was studied together with two further strains, which were also isolated from ash plants in 2017. *Bacillus velezensis* A4P130 was characterized as a typical antagonistic strain with a high ability to inhibit the growth of the pathogen *H. fraxineus* in cocultivation assays [18]. *A. altamirensis* C2P003 as well as *L. fraxinea* D4P002 showed no antagonistic activity against *H. fraxineus* [18]. However, both strains D4P002 and C2P003 were found to have significantly higher abundance (>20-fold) in tolerant ash trees and were conclusively specific for the microbiome of *F. excelsior* plants tolerant to ash dieback.

**Figure 4:**
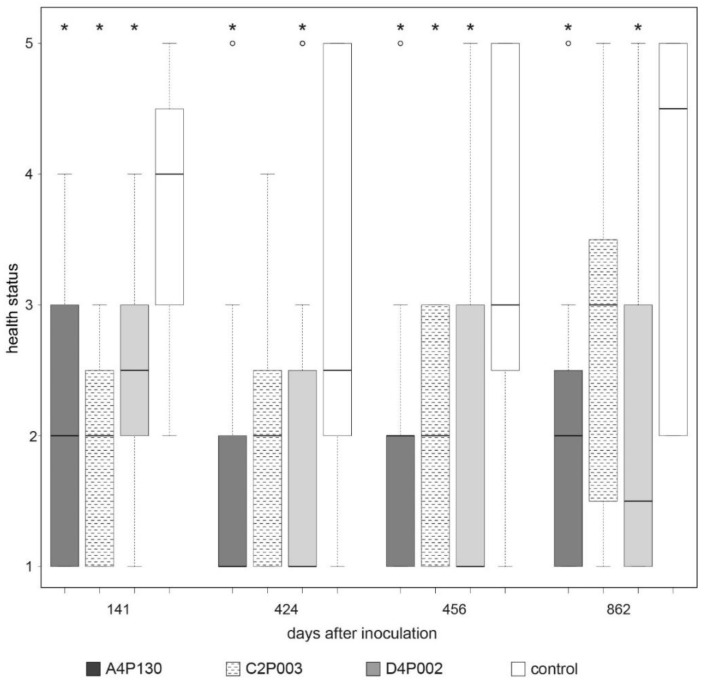
Impacts of inoculation with *A. altamirensis* C2P003, *B. velezensis* A4P130 and *L. fraxinea* D4P002 on ash seedlings. The effects on ash dieback were monitored by the health status of *H. fraxineus*-infected seedlings. Health status and ash dieback symptoms were estimated as described by Peters et al. [80]. Bonitur scale: 1: healthy, 2: 10–25% leaf damage, 3: 26–60% leaf damage, 4: 61-99 leaf damage, 5: dead plants. Stars indicate significant differences compared to the control (*p* < 0.05).

The three strains were applied for an inoculation test with ash seedlings. To simulate natural conditions of *H. fraxineus* infection, the seedlings were exposed to the pathogen via application of infected leaf petioles [79] four weeks after inoculation with the bacterial strains. This resulted in the first dieback symptoms in mid-August, such as leaves with dark patches and withered leaves [15]. The effect of the inoculation was monitored over a period of three years. In September of the first year (141 days after inoculation), all three strains showed a significant positive effect on plant health (Figure 4 and Figure S2). The strongest impact was assessed for C2P003. In the following year, new dieback infection symptoms were rarely observed. The majority of the control plants, which were still weakened by the primary infection, showed growth depressions with nonspecific leaf damage. Despite the lack of infection, there was again a distinct effect of the inoculation; however, in contrast to A4P130 and D4P002, the impact of C2P003 was significant at only one of the two time points of examination. In the third year, a strong fructification of *H. fraxineus* was again visible on the freshly applied petioles, suggesting a high likelihood of infection. Under these conditions, inoculation with the bacterial strains maintained their advantage over the controls through stronger growth and healthier leaves until the end of the vegetation period (Figure 4). The effects of inoculation with A4130 and D4P002 were significant across all three years of the plant trial [19]. Inoculation with the strain C2P003 also resulted in a distinct effect on the plant health of the ash seedlings, but at two time points this effect was not significant (Figure 4).

**Figure 5:**
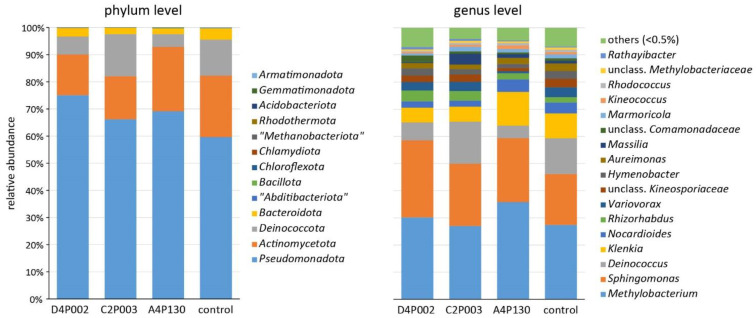
Composition of the bacterial microbiome of ash seedlings inoculated with *A. altamirensis* C2P003, *B. velezensis* A4P130 and *L. fraxinea* D4P002 as well as control plants. Two different taxonomic levels are demonstrated.

**Figure 6:**
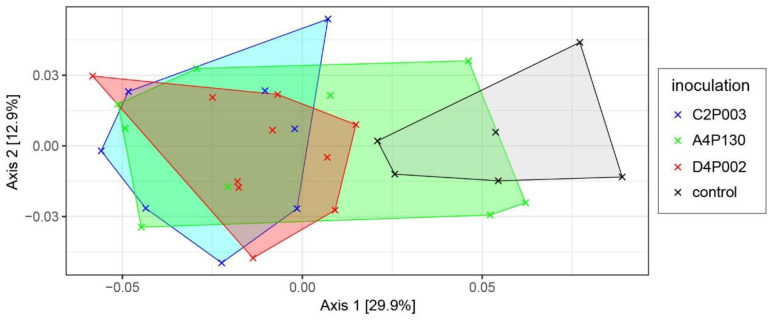
Ordination plot visualizing the bacterial microbiome composition of ash seedlings inoculated with *A. altamirensis* C2P003, *B. velezensis* A4P130 and *L. fraxinea* D4P002 as well as control plants. A principle coordinate analysis (PCoA) was applied based on the weighted UniFraq distance matrix.

In the second year (15 months after inoculation), ash leaves were sampled to compare the microbiomes of the three inoculated and the control variants. Amplicon sequencing revealed a complex bacterial community with 13 phyla dominated by *Pseudomonadota* (average abundance across all treatments of 68.5%), *Actinomycetota* (19.0%), *Deinococcota* (9.5%) and *Bacteroidota* (2.8%). An overview about the taxonomic composition of the bacterial microbiome is shown in Figure 5. At the phylum level, *Pseudomonadota* tended to be less abundant in the control plants; however, the difference was not significant. The genera *Methylobacterium*, *Sphingomonas*, *Deinococcus* and *Klenkia* dominated the bacterial microbiome and had comparable abundances in all variants. Alpha-diversity estimates (Shannon and InvSimpson index) showed significantly lower values for the microbiomes of trees inoculated with C2P003 or D4P002 (Shannon: 4.75 ± 0.34/4.94 ± 0.25; mean ± SD) as compared to the control (5.34 ± 0.26; *p* < 0.02). The variance of the bacterial microbiome composition of the ash plants was studied by a principle coordinate analysis (Figure 6). As shown in the ordination plot, the first axis clearly differentiated the plants inoculated with C2P003 and D4P002 from the control. Correspondingly, ANOSIM and PERMANOVA analyses revealed significant differences between C2P003 and D4P002 vs. the control (R = 0.47/0.42, *p* < 0.003; R^2^ = 0.27/0.24, *p* = 0.006). In contrast, the microbiome composition of A4P130-inoculated plants did not show significant differences from that of the control plants.

Altogether, the strains specifically found on tolerant ash trees were able to trigger a shift in the bacterial microbiome more than one year after the treatment. This finding is consistent with several recent studies describing that inoculation with individual bacterial strains resulted in changes in the plant microbiome that were associated with improved plant health [81,82,83,84,85]. Berg et al. [86] reviewed the effects of microbial inoculants on the indigenous plant microbiome and suggested microbiome modulations as a novel and efficient mode of action for microbial inoculants that can also be mediated via the plant.

In contrast to C2P003 and D4P002, the antagonistic strain *B. velezensis* A4P130 was not able to modify the microbiome composition, which fits well with data on the comprehensively studied biocontrol strain *B. velezensis* FZB42 [87]. Comparable to A4P130, the treatment of lettuce with FZB42 led to improved plant performance, but a lasting shift of the microbiome could not be observed [88]. In this case, the antagonistic capabilities of A4P130 were suggested for the health-protecting effects.

## 3. Materials and Methods

### 3.1. Isolation and Classification of Bacteria

*A. altamirensis* isolates were obtained during a sampling campaign of tolerant and susceptible ash trees conducted in July 2017 in a forest area of Northeast Germany with severe infestation of *H. fraxineus* [18]. All isolates originated from two tolerant trees without visible symptoms that were located in the district Pennin on plot C (54°15′ N, 13°01′ E) and a susceptible tree located in the district Lendershagen on plot A (54°14′ N, 12°51′ E). The strains were isolated from compound leaves using a method allowing the cultivation of both epi- and endophytic bacteria.

Classification was based on MALDI-TOF MS. Isolates that could not be unambiguously identified by the Bruker database were assigned by the sequencing of almost the complete 16S rRNA gene [18].

### 3.2. Sequencing of the Ribosomal Intergenic Spacer (IGS) Region

The phylogenetic variability of the selected *Aureimonas* strains was studied by sequencing the 16S–23S rRNA gene intergenic spacer region. Total DNA extraction followed the procedure of Ulrich et al. [89]. Amplification with the primers 1492f and 115r and subsequent Sanger sequencing were performed as described by Tokajian et al. [90].

### 3.3. Genome Sequencing

Cells of the *A. altamirensis* strain C2P003 were cultured in R2 broth for two days at 25 °C and washed two times with 0.3% NaCl. Genomic DNA was extracted by the Genomic-Tip 20 Kit (Qiagen, Hilden, Germany) as described previously [19]. The fragment size, quantity and quality of DNA were assessed on a 1% agarose gel and with a NanoDrop ND-1000 spectrophotometer (Thermo Scientific, Waltham, MA, USA). DNA was sequenced using the Pacific Biosciences (PacBio) RS II sequencing platform at Eurofins Genomics (Konstanz, Germany). Sequence reads were de novo assembled using the PacBio hierarchical genome assembly process (HGAP4). The assembling resulted in one contig with an average genome coverage of 124×. The genome sequence was circulated with the Circlator v. 1.5.5 [91].

### 3.4. Phylogenetic and Genome Analyses

The genome of *A. altamirensis* C2P003 was annotated using RAST server version 2.0 [92] and by the NCBI prokaryotic genome annotation pipeline [93]. The RAST platform was also used for comparisons with genomes of the *A. altamirensis* strains DSM 21988 and ON-56566 as well as *Aureimonas* sp. OT7 to avoid bias by different annotation systems. The calculation of orthologous genes was based on the predicted coding sequences with an identity of more than 70% at the amino acid level. A Venn diagram was generated using the R package VennDiagram [94].

For phylogenetic analysis, 16S rRNA gene sequences from closely related strains and species were aligned using the ClustalW algorithm with MEGA X [95], resulting in an alignment of 1386 nt. The phylogenetic tree was constructed using the maximum-likelihood algorithm based on evolutionary distances of the Tamura 3-parameter model (+G+I). In the same way, the isolate group of *A. altamirensis* was analysed using an alignment of IGS sequences comprising 950 nt. The maximum likelihood tree was calculated using distances of the Hasegawa-Kishino-Yano model (+G). The phylogenomic analysis based on core genome phylogeny [96] was performed as described by Ulrich et al. [50]. A total of 120 bacterial core marker genes were used to form a concatenated amino acid sequence alignment, which was used to calculate a maximum-likelihood tree (LG substitution model with F+G+I) with MEGA. The calculation of the ANI and dDDH values between C2P003 and the type strain of *A. altamirensis* DSM 21988 was performed as described previously [19].

### 3.5. Assessment of A. altamirensis C2P003 for Human Pathogenicity

The possible pathogenicity of C2P003 was tested by different approaches allowing a differentiation between pathogenic and nonpathogenic strains. First, C2P003 cells were plated on Columbia blood agar (Oxoid-Thermo Fisher Scientific, Germany) and incubated at 37 °C and 5% CO_2_. Growth at 37 °C is a simple indication of pathogenicity [97] and has been demonstrated for a clinical *A. altamirensis* strain [34]. A second approach consisted of checking the activity of extracellular DNase, which is involved in the dissemination of bacteria [98]. Third, haemolytic activity as a possible indication of pathogenicity was tested via incubation on Columbia blood agar as described by the manufacturer.

### 3.6. Inoculation Test on Ash Seedlings

To study the effect of antagonistic bacteria on ash dieback, ash seedlings with a height of approximately 15 cm were inoculated with the three strains *A. altamirensis* C2P003, *B. velezensis* A4P130 and *L. fraxinea* D4P002 at the beginning of June, 2019. For preparation of the inoculum, the bacteria were grown overnight in R2 broth at 22 °C with gentle shaking at 200 rpm. Cells were collected by centrifugation at 5000× *g* for 5 min, washed with 1/4 Ringer solution and finally resuspended in 20 mL 1/4 Ringer solution. The cell density of the inocula was adjusted to 1 × 10^8^ cells mL^−1^. The inoculation was performed by thoroughly dipping the seedlings in the inoculum for approximately 1 min. Control plants were immersed in the same manner in 1/4 Ringer solution. Afterwards, the seedlings were potted into 3 L plastic containers with potting soil and covered with glassine bags for 7 days to maintain high humidity. After 2 weeks, the plants were transferred to the nursery under controlled irrigation and shade. Infection with *H. fraxineus* was initiated four weeks after inoculation as well as in June of the following two years by application of infected ash leaf petioles at the bottom of the plant containers. The seedlings of each treatment (*n* = 12) were monitored for their health status based on a 5-point Bonitur scale modified according to Peters et al. [80] over a period of three years. An analysis of significant differences between inoculated and control plants was performed using the Kruskal-Wallis test followed by Dunn’s test of multiple comparisons using rank sums as implemented in R version 4.1.2, library dunn.test [99]. Fifteen months after inoculation, the seedlings were sampled for microbiome analysis. Pieces of all parts of compound leaves were taken from each of the plants and stored at −20 °C until further processing.

### 3.7. Microbiome Analysis

Total DNA was extracted from 100 mg of the ground plant material using the DNeasy Plant Mini Kit as described by Ulrich et al. [18]. PCR amplification was performed with primers 799F and 1115R which exclude the chloroplast and mitochondrial DNA of the host plant. Library preparation and 300-bp paired-end sequencing on an Illumina MiSeq was performed at LGC Genomics (Berlin, Germany). The sequence reads were processed using the DADA2 pipeline [100]. After quality filtering and the removing of potential chimaeras, residual plastid and mitochondrial sequences from ash trees were excluded from the dataset, which resulted in 2,987,432 high-quality reads. Statistical analyses were performed using the phyloseq, vegan, metagMisc, decipher, phangorn and ggplot2 packages in R. To estimate alpha diversity, the Shannon and InvSimpson indices were calculated. Differences in the bacterial microbiome composition were visualized by applying a principal coordinate analysis (PCoA) based on weighted UniFraq distances of the ASVs. Significant differences between the bacterial communities were tested using Analysis of Similarity (anosim) and PERMANOVA (adonis).

## 4. Conclusions

The studied group of *A. altamirensis* strains which were specifically found on ash trees tolerant to *H. fraxineus* infection represents the first evidence of the species as a plant-associated bacterium. A phylogenetic analysis resulted in a clear distinction from previously described clinical isolates of the species. A functional genome analysis of the representative strain C2P003 revealed the presence of many genes necessary for successful colonization of the phyllosphere and/or the endosphere of plants. Although C2P003 did not possess in vitro antagonistic activity against the fungal pathogen *H. fraxineus*, an inoculation experiment on ash seedlings demonstrated the plant health-protecting effect of C2P003, which was accompanied by a shift in the leaf microbiome one year after inoculation. It suggests that the strain C2P003 may suppress *H. fraxineus* via colonization resistance or indirectly by changing the microbiome composition.

## Figures and Tables

**Table 1:** Genome annotation statistics of the *Aureimonas altamirensis* strains C2P003, DSM 21988, ON-56566 and OT7.

	C2P003	DSM 21988	ON-56566	OT7
Contigs	1	11	13	1
Genome size (bp)	4,592,981	4,190,965	4,200,047	4,181,223
G+C content (%)	64.3	64.8	65.2	65.0
Number of protein-coding genes ^1^	4289	3967	3976	3871
rRNAs 16S (total)	3 (9)	2 (10)	1 (5)	3 (9)
tRNAs	50	46	48	51

^1^ The data were derived from the NCBI and IMG databases, and the accession numbers are CP089311, 2585427611, 2627854086 and CP062167, respectively.

**Table 2:** Summary of protein-coding genes involved in host colonization and stress adaptation in the genome of *A. altamirensis* C2P003 and corresponding genes in the genomes of DSM 21988 and ON-56566.

Category/Subsystem	Number of Genes		
	C2P003	DSM 21988	ON-56566
Capsular and extracellular polysaccharides	8	8	7
Membrane Transport			
Protein secretion system, Type I	3	3	0
Protein secretion system, Type IV	45	9	35
Protein secretion system, Type VII	3	3	0
Stress response: Osmotic stress			
Osmoprotectant ABC transporter YehZYXW of *Enterobacteriales*	4	4	4
Synthesis of osmoregulated periplasmic glucans	3	3	3
Choline and betaine uptake and betaine biosynthesis	9	9	9
Stress response: Oxidative stress			
Oxidative stress	7	8	7
Glutathione: Biosynthesis and gamma-glutamyl cycle	6	3	5
Glutathione: Nonredox reactions	9	9	9
Glutathione: Redox cycle	3	3	3
Resistance to toxic compounds			
Copper homeostasis/tolerance	8	10	11
Cobalt-zinc-cadmium resistance	8	8	8
Resistance to chromium compounds	1	1	1
Resistance to antibiotics			
Resistance to fluoroquinolones	2	2	2
Beta-lactamase	1	1	1

**Table 3:** Predicted plant and microbial cell wall-degrading enzymes of strains *A. altamirensis* C2P003, DSM 21988 and ON-56566.

CAZy Family	Substrate	Annotation	Enzyme Code	Number of Genes		
				C2P003	DSM 21988	ON-56566
CE4	Peptidoglycans	Peptidoglycan *N*-acetylglucosamine deacetylase	EC 3.5.1.-	1	0	0
CE4	Polysaccharides	Polysaccharide deacetylase		2	0	0
	Peptidoglycans	D-alanyl-D-alanine carboxypeptidase	EC 3.4.16.4	4	4	4
GH73	Peptidoglycans	*N*-acetylmuramoyl-L-alanine amidase	EC 3.5.1.28	1	1	1
CE9	Polysaccharides/Chitooligosaccharides	*N*-acetylglucosamine-6-phosphate deacetylase	EC 3.5.1.25	1	1	1
	Polysaccharides	Glucosamine-6-phosphate deaminase	EC 3.5.99.6	1	1	1
GH13	Polysaccharides	α-amylase	EC 3.2.1.1	1	1	1
GH15	Polysaccharides	Glucoamylase	EC 3.2.1.3	2	2	1
GT35	Polysaccharides	Glycogen phosphorylase	EC 2.4.1.1	1	1	1
GH43	Hemicellulose	α-L-arabinofuranosidase	EC 3.2.1.55	1	0	1
GH9	Cellulose	β-1,4-glucanase	EC 3.2.1.4	1	1	1
GH28	Polysaccharides/Pectin	Pectin degradation protein		1	1	0
GH24	Peptidoglycans/Chitin	Phage lysozyme R	EC 3.2.1.17	2	1	1
GH73	Peptidoglycans/Chitin	β-*N*-acetylglucosaminidase	EC 3.2.1.52	1	1	1
GH15	Polysaccharides	Glucan 1,4-α-glucosidase	EC 3.2.1.3	1	1	0
GH16	Cellulose, Hemicellulose	Endo-β-1,3-1,4 glucanase (licheninase)	EC 3.2.1.73	1	1	1

## Data Availability

The genome sequence of the strain C2P003 was deposited in the GenBank database under the accession no. CP089311. The paired sequence reads generated for the microbiome analysis were deposited in the Sequence Read Archive under accession number PRJNA809658.

## Supplementary Materials

The following supporting information can be downloaded at: https://www.mdpi.com/article/10.3390/plants11243487/s1, Figure S1: Phylogenomic tree showing the position of *A. altamirensis* C2P003 among other *A. altamirensis* strains and reference strains of related *Aureimonas* species. Figure S2: Ash seedlings inoculated with *B. velezensis* A4P130 (A), *A. altamirensis* C2P003 (B) and *L. fraxinea* D4P002 (C) as well as control plants (D). Plant health was evaluated 141 days after inoculation. Table S1: Protein-encoding genes involved in host colonization and stress adaptation in the genome of *A. altamirensis* C2P003. Table S2: Protein-encoding genes involved in the degradation of plant and microbe cell walls in the genome of *A. altamirensis* C2P003.
