# Supplementary material for: Genomic Characterization of Aureimonas altamirensis C2P003—A Specific Member of the Microbiome of Fraxinus excelsior Trees Tolerant to Ash Dieback
Source: Plants (Basel). 2022 Dec 13;11(24):3487. doi: 10.3390/plants11243487 (PMC9781493; doi:10.3390/plants11243487)
Supplement: Supplementary file 1 [file plants-11-03487-s001.zip › supplementary files/Fig. S1 Phylogenomic tree.pdf]

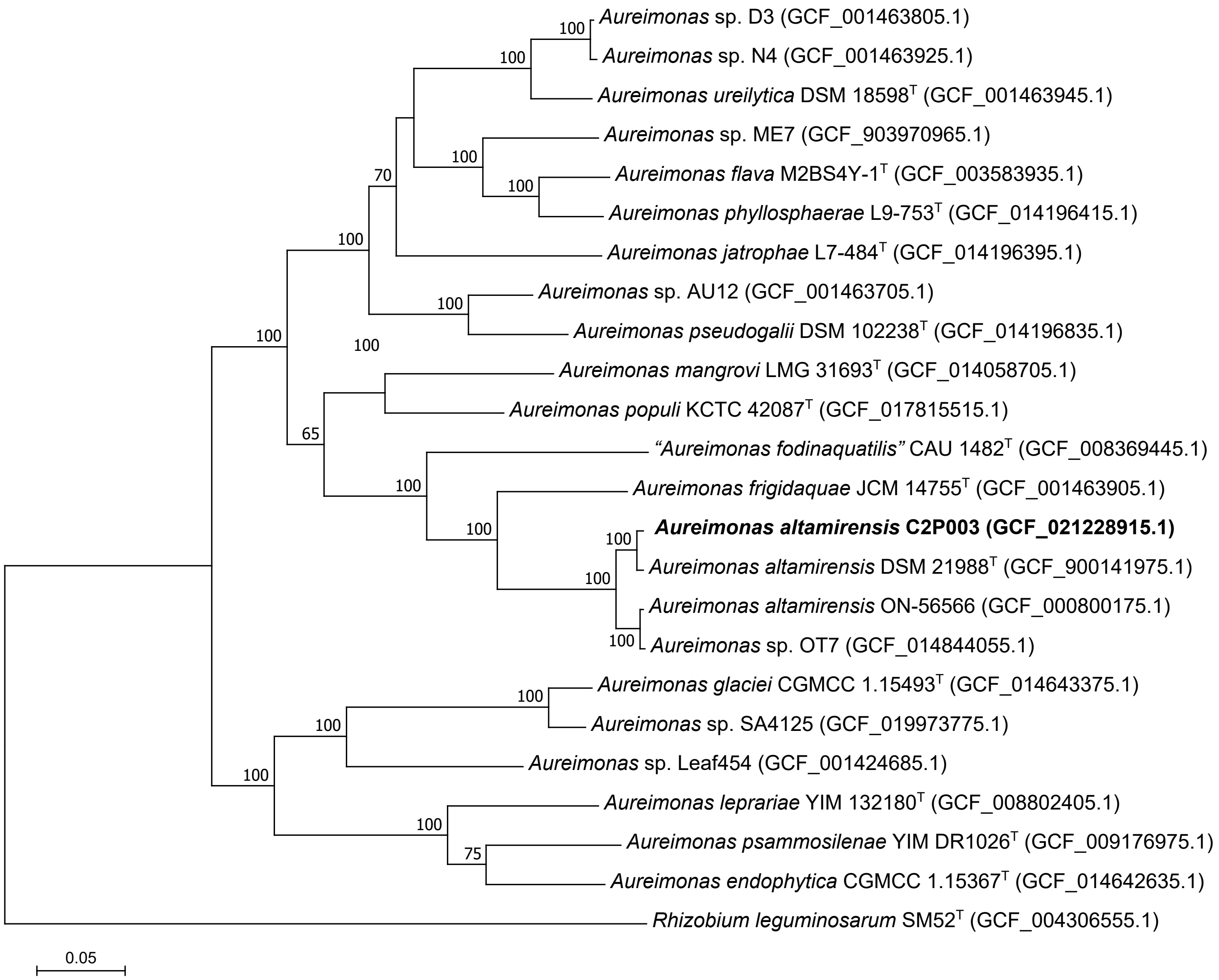

Figure S1. Phylogenomic tree showing the relationship of of *A. altamirensis* C2P003 among closely related strains and species of the genus *Aureimonas*. *Rhizobium leguminosarum* SM52 was used as the outgroup. The maximum-likelihood tree is based on 120 concatenated core marker proteins. Numbers at branch nodes refer to bootstrap values > 50 %. Assembly accession numbers are indicated in brackets. Bar: amino acid substitutions per position.
