# Supplementary material for: Genomic Characterization of Aureimonas altamirensis C2P003—A Specific Member of the Microbiome of Fraxinus excelsior Trees Tolerant to Ash Dieback
Source: Plants (Basel). 2022 Dec 13;11(24):3487. doi: 10.3390/plants11243487 (PMC9781493; doi:10.3390/plants11243487)
Supplement: Supplementary file 1 [file plants-11-03487-s001.zip › supplementary files/Supplement tables.docx]

**Table S1.** Protein-coding genes involved in host colonization and stress adaptation in the genome of *A. altamirensis* C2P003.

| Category/subsystem | Location in  C2P003 | Enyme code | Function | Presence of the gene in  DSM 21988 ON-56566  (number of copies) | |
| --- | --- | --- | --- | --- | --- |
| Capsular and extracellular polysaccharides | 520.548 - 519.658 | EC 2.-.-.- | dTDP-rhamnosyl transferase RfbF | 1 | - |
|  | 522.581 - 521.697 | EC 1.1.1.133 | dTDP-4-dehydrorhamnose reductase | 1 | 1 |
|  | 523.642 - 522.578 | EC 4.2.1.46 | dTDP-glucose 4,6-dehydratase | 1 | 1 |
|  | 524.212 - 523.646 | EC 5.1.3.13 | dTDP-4-dehydrorhamnose 3,5-epimerase | 1 | 1 |
|  | 524.405 - 525.289 | EC 2.7.7.24 | Glucose-1-phosphate thymidylyltransferase | 1 | 1 |
|  | 1.413.698 - 1.412.709 2.002.742 - 2.001.786 2.238.655 - 2.237.780 | EC 5.1.3.2 | UDP-glucose 4-epimerase | 3 | 3 |
| Membrane Transport:  Protein secretion system, Type I | 957.754 - 959.247 |  | Type I secretion system, outer membrane component LapE | 1 | - |
|  | 959.393 - 961.459 |  | Type I secretion system ATPase, LssB family LapB | 1 | - |
|  | 961.456 - 962.646 |  | Type I secretion membrane fusion protein, HlyD family @ Type I secretion system, membrane fusion protein LapC | 1 | - |
| Protein and nucleoprotein secretion system, Type IV | 55242 - 54049  789.447 - 790.592  2.861.729 - 2.862.877  4.093.423 - 4.092.287  4.333.188 - 4.332.052 |  | Conjugative transfer protein TrbI | 1 | 4 |
|  | 56222 - 55239  788.431 - 789.450  2.860.710 - 2.861.732  4.094.436 - 4.093.420  4.334.180 - 4.333.185 |  | Conjugative transfer protein TrbG | 1 | 4 |
|  | 56911 - 56219  787.745 - 788.434  2.860.024 - 2.860.713  4.095.122 - 4.094.433  4.334.866 - 4.334.177 |  | Conjugative transfer protein TrbF | 1 | 4 |
|  | 58242 - 56908  786.405 - 787.748  2.858.681 - 2.860.027  4.096.486 - 4.095.119  4.336.221 - 4.334.863 |  | Conjugative transfer protein TrbL | 1 | 4 |
|  | 59315 – 58554  785.317 - 786.099  2.857.593 - 2.858.369  4.097.563 - 4.096.796  4.337.281 - 4.336.505 |  | Conjugative transfer protein TrbJ | 1 | 4 |
|  | 61750 - 59312  782.864 - 785.320  2.855.140 - 2.857.596  4.100.070 - 4.097.560  4.339.770 - 4.337.278 |  | Conjugative transfer protein TrbE | 1 | 5 |
|  | 62043 - 61762  782.572 - 782.853  2.854.846 - 2.855.127  4.100.362 - 4.100.081  4.340.062 - 4.339.781 |  | Conjugative transfer protein TrbD | 1 | 4 |
|  | 62375 - 62043  782.240 - 782.572  2.854.514 - 2.854.846  4.100.748 - 4.100.362  4.340.394 - 4.340.062 |  | Conjugative transfer protein TrbC | 1 | 1 |
|  | 63355 - 62372  781.263 - 782.243  2.853.537 - 2.854.517  4.101.725 - 4.100.745  4.341.374 - 4.340.391 |  | Conjugative transfer protein TrbB | 1 | 5 |
| Protein secretion system,Type VII | 2.243.786 - 2.241.444 |  | Sigma-fimbriae usher protein | 1 | - |
|  | 2.244.296 - 2.243.871 |  | Sigma-fimbriae chaperone protein | 1 | - |
|  | 2.244.818 - 2.244.324 |  | Sigma-fimbriae tip adhesin | 1 | - |
| Osmotic stress response:  Osmoprotectant ABC transporter YehZYXW of En-terobacteriales | 3.973.452 - 3.972.697 |  | Osmoprotectant ABC transporter inner membrane protein YehW | 1 | 1 |
|  | 3.974.387 - 3.973.449 |  | Osmoprotectant ABC transporter ATP-binding subunit YehX | 1 | 1 |
|  | 3.975.544 - 3.974.384 |  | Osmoprotectant ABC transporter permease protein YehY | 1 | 1 |
|  | 3.976.477 - 3.975.566 |  | Osmoprotectant ABC transporter binding protein YehZ | 1 | 1 |
| Synthesis of osmoregulated periplasmic glucans | 626.796 - 628.298 |  | Glucans biosynthesis protein D precursor | 1 | 1 |
|  | 4.454.095 - 4.455.705 |  | Glucans biosynthesis protein G precursor | 1 | 1 |
|  | 4.457.899 - 4.459.110 |  | OpgC protein | 1 | 1 |
| Choline and betaine uptake and betaine biosynthesis | 254.556 - 253.045 | EC 3.1.6.6 | Choline-sulfatase | 1 | 1 |
|  | 460.751 - 461.512 | TC 3.A.1.12.1 | L-proline glycine betaine ABC transport system permease protein ProW | 1 | 1 |
|  | 2.102.825 - 2.104.933 | EC 1.2.1.8 | Betaine aldehyde dehydrogenase | 1 | 1 |
|  | 2.104.944 - 2.106.605 | EC 1.1.99.1 | Choline dehydrogenase | 1 | 1 |
|  | 2.130.195 - 2.129.629  2.133.157 - 2.130.188  2.133.456 - 2.133.154  2.134.729 - 2.133.473 | EC 1.5.3.1 | Sarcosine oxidase gamma subunit | 4 | 4 |
|  | 2.617.555 - 2.615.561 |  | High-affinity choline uptake protein BetT | 1 | 1 |
| Oxidative stress response:  Oxidative stress | 6.808 - 6.356 |  | Iron-responsive regulator Irr | 1 | 1 |
|  | 19.350 - 18.637  1.727.137 - 1.727.739 | EC 1.15.1.1 | Superoxide dismutase [Mn] | 2 | 2 |
|  | 311.685 - 312.122 |  | Redox-sensitive transcriptional activator SoxR | - | - |
|  | 1.470.085 - 1.467.491 | EC 2.7.3.- | Phytochrome, two-component sensor histidine kinase | 2 | 2 |
|  | 3.390.454 - 3.389.900 |  | Hydrogen peroxide-inducible genes activator | 2 | 1 |
|  | 4.549.160 - 4.549.897 |  | Transcriptional regulator, Crp/Fnr family | 1 | 1 |
| Glutathione: Biosynthesis and gamma-glutamyl cycle | 1.158.605 - 1.160.176  2.589.496 - 2.587.754  3.017.122 - 3.015.530  3.350.390 - 3.351.976 | EC 2.3.2.2  EC 3.4.19.13 | Gamma-glutamyltranspeptidase) @ Glutathione hydrolase | 1 | 3 |
|  | 1.512.093 - 1.510.720 | EC 6.3.2.2 | Glutamate-cysteine ligase | 1 | 1 |
|  | 4.491.239 - 4.492.177 | EC 6.3.2.3 | Glutathione synthetase | 1 | 1 |
| Glutathione: Nonredox reactions | 281.179 - 280.511 |  | Glutathione S-transferase family protein | 1 | 1 |
|  | 1.000.412 - 1.001.182 | EC 3.1.2.6 | Hydroxyacylglutathione hydrolase | 1 | 1 |
|  | 1.076.833 - 1.076.171  1.424.892 - 1.425.518  1.458.734 - 1.458.120  2.955.015 - 2.954.143  4.063.019 - 4.064.020 | EC 2.5.1.18 | Glutathione S-transferase | 5 | 5 |
|  | 2.257.423 - 2.257.863  2.697.928 - 2.697.530 | EC 4.4.1.5  EC 4.4.1.5 | Lactoylglutathione lyase | 2 | 2 |
| Glutathione: Redox cycle | 1.024.226 - 1.024.363 |  | Glutaredoxin 3 (Grx2) | 1 | 1 |
|  | 3.309.807 - 3.308.13 | EC 1.8.1.7 | Glutathione reductase | 1 | 1 |
|  | 3.595.383 - 3.595.730 |  | Uncharacterized monothiol glutaredoxin ycf64-like | 1 | 1 |
| Resistance to toxic compounds: Copper homeostasis / tolerance | 492.805 - 493.248 |  | Cu(I)-responsive transcriptional regulator | 1 | 1 |
|  | 1.190.604 – 1.192.877 | EC 3.6.3.3  EC 3.6.3.5  EC 3.6.3.4 | Lead, cadmium, zinc and mercury transporting ATPase Copper-translocating P-type ATPase | 4 | 4 |
|  | 1.070.057 - 1.071.247  2.073.922 - 2.074.512  3.243.265 - 3.244.473 |  | Multidrug resistance transporter, Bcr/CflA family | 2 | 3 |
|  | 1.273.949 - 1.274.989 |  | Magnesium and cobalt efflux protein CorC | 1 | 1 |
|  | 1.274.986 - 1.276.563 |  | Apolipoprotein N-acyltransferase / Copper homeostasis protein CutE | 1 | 1 |
|  | 3.677.292 - 3.675.307 |  | Cytochrome c heme lyase subunit CcmF | 1 | 1 |
| Cobalt-zinc-cadmium resistance | 701.443 - 702.561  1.759.843 - 1.758.569 |  | Probable Co/Zn/Cd efflux system membrane fusion protein | 2 | 2 |
|  | 725.533 - 725.216 |  | Cobalt-zinc-cadmium resistance protein CzcA; Cation efflux system protein CusA | 1 | 1 |
|  | 862.083 - 861.655  1.715.224 - 1.714.799  2.927.364 - 2.926.768  3.126.492 – 3.126.100 |  | Transcriptional regulator, MerR family | 4 | 4 |
|  | 4.070.369 - 4.069.473 |  | Cobalt-zinc-cadmium resistance protein | 1 | 1 |
| Resistance to chromium compounds | 3.528.533 - 3.527.313 |  | Chromate transport protein ChrA | 1 | 1 |
| Resistance to antibiotics  Resistance to fluoroquinolones | 2.492.083 - 2.494.851 | EC 5.99.1.3 | DNA gyrase subunit A | 1 | 1 |
|  | 4.538.669 - 4.536.234 | EC 5.99.1.3 | DNA gyrase subunit B | 1 | 1 |
| Beta-lactamase | 2.698.431 - 2.697.991 |  | Metal-dependent hydrolases of the beta-lactamase superfamily I; PhnP protein | 1 | 1 |

**Table S2.** Protein-coding genes involved in the degradation of plant and microbe cell walls in the genome of *A. altamirensis* C2P003.

| CAZy family | Substrate | Annotation | Enyme code | Location of the gene in  C2P003 | Presence of the gene in  DSM 21988 ON-56566  (number of copies) | |
| --- | --- | --- | --- | --- | --- | --- |
| CE4 | Peptidoglycanes | Peptidoglycan *N*-acetylglucosamine deacetylase | EC 3.5.1.- | 134.235 - 133.519 | - | - |
| CE4 | Polysaccharides | Polysaccharide deacetylase |  | 862.219 - 863.130 | - | - |
| CE4 | Polysaccharides | Putative polysaccharide deacetylase |  | 2.443.866 - 2.444.789 | - | - |
|  | Peptidoglycanes | D-alanyl-D-alanine carboxypeptidase | EC 3.4.16.4 | 1.376.554 - 1.377.687  2.239.575 - 2.238.700  2.704.082 - 2.702.886  3.120.816 - 3.122.387 | 4 | 4 |
| GH73 | Peptidoglycanes | *N*-acetylmuramoyl-L-alanine amidase | EC 3.5.1.28 | 520.548 - 519.658 | 1 | 1 |
| CE9 | Polysaccharides / Chitooligosaccharides | *N*-acetylglucosamine-6-phosphate deacetylase | EC 3.5.1.25 | 2.886.802 - 2.885.636 | 1 | 1 |
|  | Polysaccharides | Glucosamine-6-phosphate deaminase | EC 3.5.99.6 | 2.887.827 - 2.886.802 | 1 | 1 |
| GH13 | Polysaccharides | α-amylase | EC 3.2.1.1 | 1.366.594 - 1.368.627 | 1 | 1 |
| GH15 | Polysaccharides | Glucoamylase | EC 3.2.1.3 | 3.329.489 - 3.331.321  3.964.358 - 3.966.220 | 2 | 1 |
| GT35 | Polysaccharides | Glycogen phosphorylase | EC 2.4.1.1 | 1.594.882 - 1.592.369 | 1 | 1 |
| GH43 | Hemicellulose | α-L-arabinofuranosidase | EC 3.2.1.55 | 190.809 - 189.298 | - | 1 |
| GH9 | Cellulose | β-1,4-glucanase | EC 3.2.1.4 | 486.681 - 487.727 | 1 | 1 |
| GH28 | Polysaccharides / Pectin | Pectin degradation protein |  | 1.164.902 - 1.164.549 | 1 | - |
| GH24 | Peptidoglycanes / Chitin | Phage lysozyme R | EC 3.2.1.17 | 1.973.607 - 1.974.371  3.684.276 - 3.683.737 | 1 | 1 |
| GH73 | Peptidoglycanes /Chitin | β-*N*-acetylglucosaminidase | EC 3.2.1.52 | 2.299.916 - 2.300.962 | 1 | 1 |
| GH15 | Polysaccharides | Glucan 1,4-α-glucosidase | EC 3.2.1.3 | 2.763.511 - 2.765.802 | 1 | - |
| GH16 | Cellulose, Hemicellulose | Endo-β-1,3-1,4 glucanase (licheninase) | EC 3.2.1.73 | 4.439.157 - 4.438.375 | 1 | 1 |
