# Supplementary figures and images for: Genomic Characterization of Aureimonas altamirensis C2P003—A Specific Member of the Microbiome of Fraxinus excelsior Trees Tolerant to Ash Dieback
Source: Plants (Basel). 2022 Dec 13;11(24):3487. doi: 10.3390/plants11243487 (PMC9781493; doi:10.3390/plants11243487)

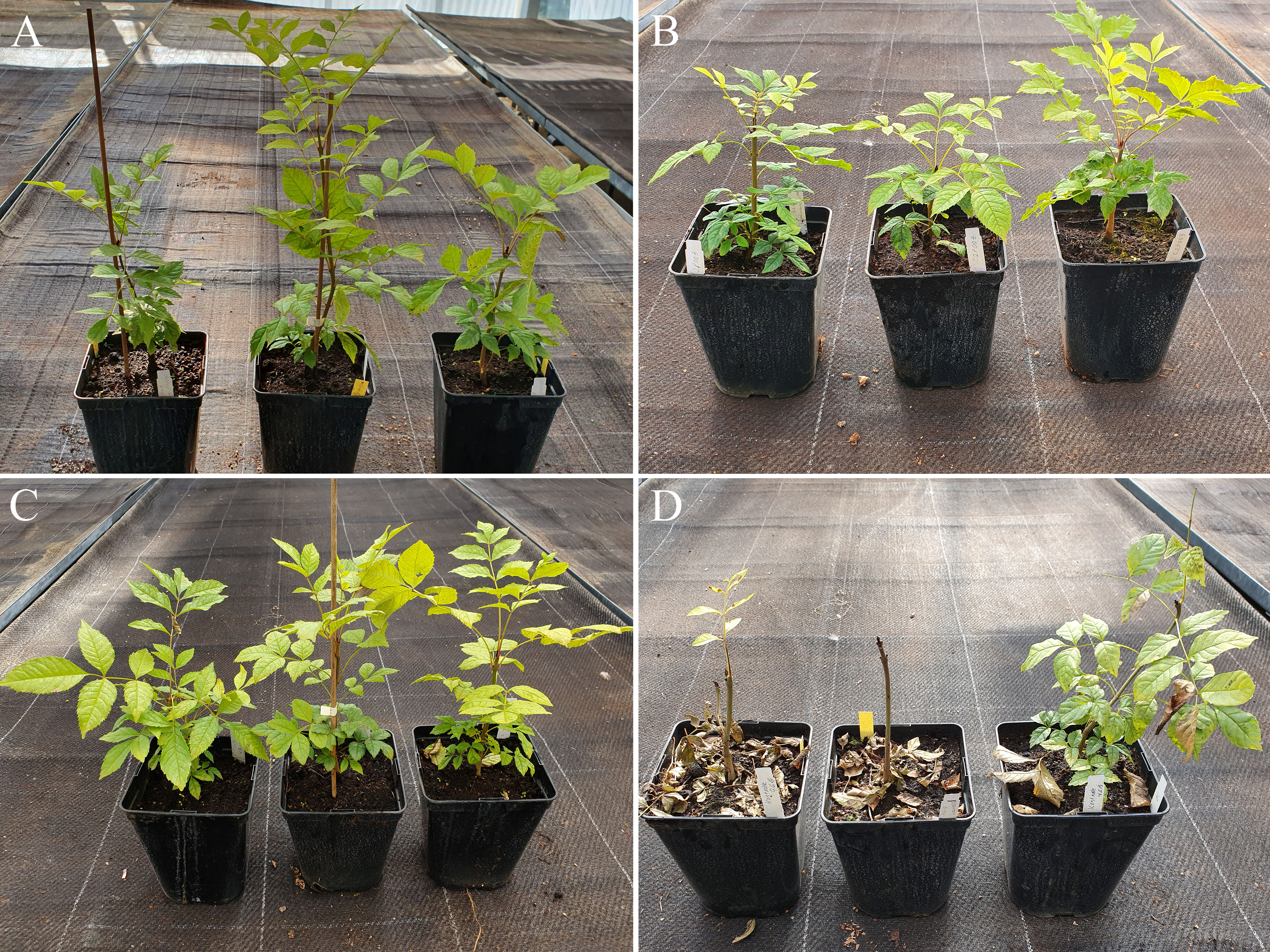

Supplement: Supplementary file 1 [file plants-11-03487-s001.zip › supplementary files/Fig. S2 plant test.jpg]
